# Systematic Investigations of the Huperzine A—Producing Endophytic Fungi of *Huperzia serrata* in China and Fermentation Optimization Using OSMAC Strategy

**DOI:** 10.3390/molecules30132704

**Published:** 2025-06-23

**Authors:** Wei Li, Zhicheng Wang, Qiuyu Zhu, Pingfang Tian

**Affiliations:** Beijing Key Laboratory of Bioprocess, College of Life Science and Technology, Beijing University of Chemical Technology, Beijing 100029, China; 2021400299@buct.edu.cn (W.L.); 2023201234@buct.edu.cn (Z.W.); 2022400332@buct.edu.cn (Q.Z.)

**Keywords:** Alzheimer’s disease, *Huperzia serrata*, huperzine A, endophytic fungus, transcriptome analysis

## Abstract

Huperzine A (HupA) can alleviate Alzheimer’s disease due to its reversible inhibition of acetylcholinesterase (AChE). The chemical synthesis and plant extraction of HupA is plagued by route complexity and resource scarcity, respectively. Although some endophytic fungi from *Huperzia serrata* can independently biosynthesize HupA, their yields are trivial. After a comprehensive investigation of HupA-producing *H. serrata* across China, we focused on the endophytic fungi from Hunan and Hubei provinces, which demonstrated high-level HupA. Morphological characteristics and internal transcriptional sequence (ITS) analysis revealed their diversity. Among the four HupA-producing endophytic fungi, *Colletotrichum kahawae* is the best-performing and was thus subjected to fermentation optimization. When its fermentation medium was supplemented with *H. serrata* flavonoids daidzein and apigenin, HupA yields reached 58.38 μg/g (dry cell weight, dcw) and 72.21 μg/g dcw, respectively. In contrast, the addition of L-lysine and *H. serrata* extracts led to yields of 50.17 μg/g dcw and 255.32 μg/g dcw, respectively. Transcriptomic analysis revealed that *H. serrata* extracts substantially upregulated the expression of HupA biosynthesis genes in *C. kahawae*. Overall, *H. serrata* extracts outperformed L-lysine, daidzein, and apigenin in boosting HupA production, as they encompass all the necessary nutrients for *C. kahawae* growth. This study not only connotes a nutritional exchange between *H. serrata* and *C. kahawae* during long-term coevolution but also offers insights for harnessing plant extracts for the overproduction of desired metabolites in endophytic fungi.

## 1. Introduction

Alzheimer’s disease (AD) is a neurodegenerative disorder characterized by memory loss and cognitive deficits [1,2]. AD is currently one of the leading causes of mortality among the elderly [3]. It is reckoned that approximately 33 million people worldwide are suffering from AD, and this number may soar to 102 million by 2050. This striking increase will pose a severe challenge to the global healthcare system. While advances in medications and early diagnostic methods have been made to cope with this crisis [2,4], there remains a need for alternative approaches, such as plant-derived medicines. Huperzine A (HupA) is a Lycopodium alkaloid that alleviates AD through the reversible inhibition of acetylcholinesterase (AChE), a critical enzyme in synaptic function [5,6]. In China, HupA has been approved as a therapeutic agent for AD, while in the United States, it serves as a dietary supplement to mitigate memory decline [7]. Although HupA is commonly extracted from *Huperzia* plants (e.g., *Huperzia serrata*) [5,8], this method is constrained by complex extraction processes and the scarcity of plant resources. In China, *H. serrata* has been viewed as an endangered species due to its slow growth and over-harvesting in recent years [5,7]. Additionally, wild-type *Huperzia* plants contain only 0.0046% to 0.0133% HupA by weight [7], meaning that large quantities of plant material are needed for extraction. Chemical synthesis involves complex technical processes and often generates by-products that complicate the purification of HupA. Moreover, chemical synthesis requires expensive reagents and catalysts [5], indicative of high costs and environmental pollution. Overall, neither plant extraction nor chemical synthesis is suitable for the sustainable production of HupA [5].

Microbial fermentation is an alternative to plant extraction and chemical synthesis. However, it is unlikely to engineer a recombinant HupA-producing strain, because the biosynthesis genes have not been completely identified. Fortunately, some endophytic fungi isolated from *H. serrata* have been shown to independently synthesize HupA [9]. In principle, HupA production can be enhanced through the precise addition of substrates or intermediate metabolites, guided by the real-time monitoring of the expression levels of key enzymes. In this context, the HupA-producing endophytic fungus can be regarded as a “grey box,” where the expression levels of these enzyme genes serve as indicators of how extracellular factors—such as carbon sources, nitrogen sources, trace elements, and dissolved oxygen—influence HupA production. Indeed, studies have shown that supplementing the fermentation broth with trace amounts of L-lysine and *H. serrata* extracts can significantly boost HupA production [10]. Apart from enhancing fermentation yields, the introduction of specific fermentation ingredients may also upregulate gene expression or even activate previously silenced genes. For instance, a hybrid molecule with phytotoxic activity was recently identified through genome mining, heterologous expression, and the One Strain Many Compounds (OSMAC) strategy [11]. In recent years, the OSMAC strategy has been implemented for activating silent genes and diversifying metabolite profiles by manipulating culture media, growth conditions, and co-cultivation methods [12,13,14,15,16,17].

Based on the aforementioned information, we conjectured that high-level production of HupA could be achieved by harnessing a best-performing endophytic fungus, coupled with the precise optimization of fermentation medium. With this in mind, in this study, fingerprint analysis was conducted to elucidate variations in HupA content among samples of *H. serrata* collected from different regions across China, including Hubei, Anhui, Hunan, Guangxi Zhuang Autonomous Region, Yunnan, and Jilin provinces. Subsequently, we investigated the endophytic fungi within the leaves of *H. serrata* collected from Hunan and Hubei provinces, focusing on those capable of producing HupA. The isolated fungal strains were then identified based on their morphological characteristics and internal transcribed spacer (ITS) sequences. After evaluating the innate HupA levels in various endophytic fungi, we selected the best-performing strain for subsequent fermentation optimization. The addition of L-lysine or *H. serrata* extracts to the fermentation broth aimed to elucidate their impact on HupA production. To gain deeper insights, we conducted both long-read and short-read RNA sequencing (RNA-seq) of *H. serrata* to construct a comprehensive transcriptome dataset. The subsequent transcriptome analysis of different tissues from *H. serrata* aimed to identify highly co-expressed biosynthetic enzymes involved in HupA synthesis. The transcriptional analysis described above may provide valuable insights into the gene flow or co-evolutionary dynamics between *H. serrata* and its endophytic fungi. Overall, this study aimed to enhance HupA production in endophytic fungi, particularly in cases where the relevant genes have yet to be fully identified.

## 2. Results

### 2.1. Fingerprint Analysis Reveals Composition and Relative Proportions

**Figure 1:**
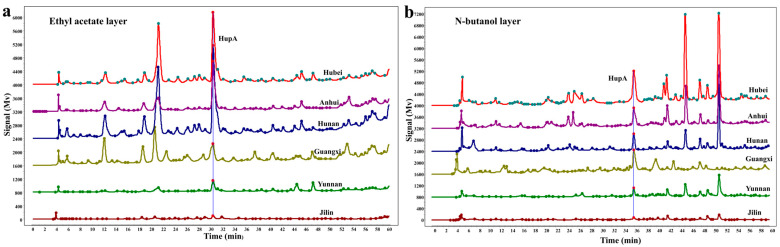
Levels of HupA in *H. serrata* samples sourced from six regions in China. (**a**). The fingerprint showing the compositional variations in *H. serrata* extracts obtained via ethyl acetate across the six distinct regions. (**b**). The fingerprint showing the compositional variations in *H. serrata* extracts obtained via n-butanol across the six distinct regions. The six regions are Huaihua City (Hunan Province), Chihe City (Guangxi Zhuang Autonomous Region), Bozhou City (Anhui Province), Kunming City (Yunnan Province), Enshi City (Hubei Province), and Jilin City (Jilin Province).

The *H. serrata* crude extracts obtained via liquid-phase gradient elution manifested overlapping peaks, which impeded accurate chromatographic analysis. To isolate compounds with varying polarities, the samples were sequentially extracted using water, petroleum ether, ethyl acetate (EtOAc), and n-butanol [18,19]. Given that EtOAc and n-butanol are particularly effective for extracting alkaloids and other bioactive fractions, these extracts were subjected to HPLC analysis to generate a chemical fingerprint. Fingerprint analysis revealed the composition and relative proportions of the bioactive compounds in *H. serrata* samples from the six different regions (Figure 1a,b). Interestingly, the extracts from Hunan, Hubei, Anhui provinces, and the Guangxi Zhuang Autonomous Region exhibited higher levels and greater diversity of bioactive compounds compared to those from Yunnan and Jilin provinces. Further analysis revealed that the peak patterns of the components from Hunan, Hubei, Anhui, and Guangxi were similar yet displayed subtle differences. In contrast, the components from Yunnan province were relatively lower in abundance, while those from Jilin province were extremely scarce. Notably, the EtOAc extracts from Hunan and Hubei provinces showed higher levels of HupA than those from other provinces (Figure 1a). Moreover, the levels of HupA in the EtOAc extracts exceeded those in the n-butanol extracts; although, the fingerprint pattern of the n-butanol extracts was more complex and diverse (Figure 1a,b). Overall, EtOAc is a medium-polarity solvent, while alkaloids are medium-polarity compounds. This similarity in polarity suggests that most alkaloids tend to concentrate in the EtOAc fraction. Conversely, n-butanol is a biphasic solvent capable of dissolving both polar substances and certain non-polar compounds, including some alkaloids.

### 2.2. Morphology of Endophytic Fungi in H. serrata

**Figure 2:**
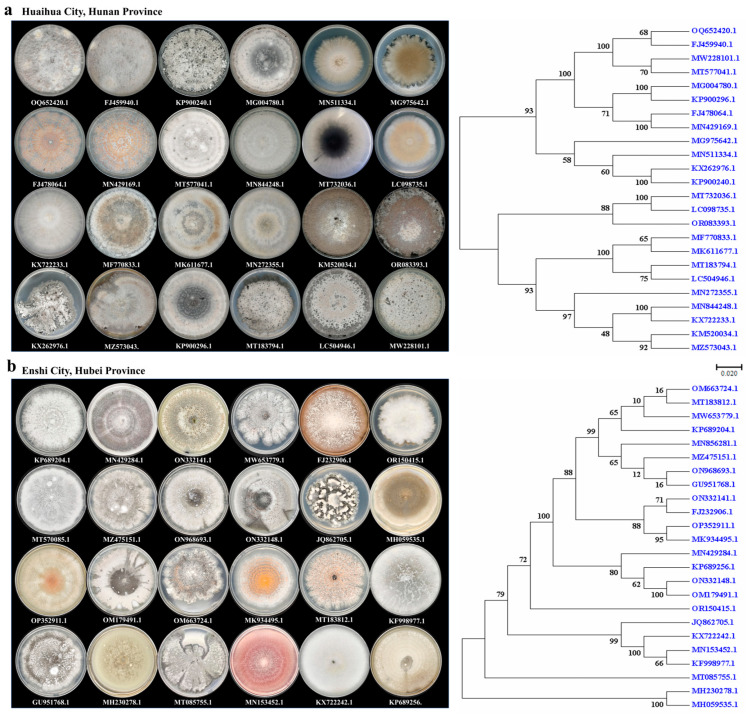
Endophytic fungi isolated from *H. serrata* in Huaihua City (Hunan Province) and Enshi City (Hubei Province). (**a**). Morphologies, growth characteristics on PDA (Potato Dextrose Agar) plates, and phylogenetic tree analysis of endophytic fungi isolated from *H. serrata* in Hunan province. (**b**). Morphologies, growth characteristics on PDA plates, and phylogenetic tree analysis of endophytic fungi isolated from *H. serrata* in Hubei province.

Given that *H. serrata* from Hunan and Hubei provinces exhibited higher levels of HupA, we proceeded to isolate and purify endophytic fungi from fresh leaf tissues. The isolated endophytic fungi displayed considerable morphological diversity, which may be attributed to various factors, such as fungal species, host developmental stage, inoculation density, and environmental conditions. A total of 24 distinct species from both provinces are shown in Figure 2a,b. The colonies of endophytic fungi manifested circular or irregular shapes, with distinct edges and either smooth or wrinkled surfaces. Notably, the same endophytic fungus exhibited variations in hyphal color across different growth stages. Microscopic analysis revealed that most mycelia were branched; although, some formed sporangia and spores [9,20].

### 2.3. Identification Based on ITS Sequences and Phylogenetic Analysis

Due to vast taxonomic diversity, it is challenging to isolate, identify, and characterize endophytic fungi. BLAST (https://blast.ncbi.nlm.nih.gov) search against GenBank using the ITS rRNA gene as a query revealed high sequence similarity among the isolates (Tables S1 and S2). A phylogenetic tree of endophytic fungi from Hubei and Hunan provinces was constructed using the neighbor-joining (NJ) method (Figure 2a,b). Upon comparing the phylogenetic trees from these two provinces, we observed that the initial eight branches of the Hubei province’s phylogenetic tree exhibited lower bootstrap values (Figure 2b, Table S2). Further analysis revealed that these eight sequences corresponded to the following species: *Colletotrichum aenigma* (OM663724.1), *Sordariomycetes* sp. (MT183812.1), *Colletotrichum siamense* (MW653779.1), *Colletotrichum gloeosporioides* (KP689204.1), *Colletotrichum* sp. LWYF83 (MT570085.1), *Colletotrichum jiangxiense* (MZ475151.1), *Colletotrichum horii* (ON968693.1), and *Colletotrichum* sp. SF3 (GU951768.1). Most of these species belong to the genus *Colletotrichum*, and their high sequence similarity likely complicates their distinction, resulting in lower bootstrap values. Additionally, *Phyllosticta capitalensis* (MT085755.1) was independently classified into a separate branch of the phylogenetic tree.

### 2.4. Isolation of Endophytic Fungi from H. serrata

Hunan and Hubei provinces, situated in the subtropical region of China, exhibit distinct ecological niches and climatic conditions. These differences likely drive variations in the ecological adaptability and physiological metabolism of endophytic fungi. In this study, a total of 30 and 58 endophytic fungi were isolated and purified from the fresh leaf tissues of *H. serrata* collected from Hunan and Hubei provinces, respectively (Tables S1 and S2). Based on ITS analysis, the endophytic fungi from Hunan province were classified into 14 genera, including *Colletotrichum*, *Whalleya*, *Phyllosticta*, *Sordariomycete*, *Annulohypoxylon*, *Xylariaceae*, *Pestalotiopsis*, *Hypoxylon*, *Nigrospora*, *Nodulisporium*, *Daldinia*, *Penicillium*, *Cladosporium*, and *Fusarium*. In contrast, those from Hubei province were categorized into nine genera: *Colletotrichum*, *Phyllosticta*, *Nigrospora*, *Paraboeremia*, *Podospora*, *Sordariomycetes*, *Xylariales*, *Fusarium*, and *Phoma*. Notably, the two provinces span five genera: *Colletotrichum*, *Phyllosticta*, *Sordariomycetes*, *Nigrospora*, and *Fusarium*, with *Colletotrichum* being the most predominant genus. Specifically, the genera *Whalleya*, *Annulohypoxylon*, *Xylariaceae*, *Pestalotiopsis*, *Hypoxylon*, *Nodulisporium*, *Daldinia*, *Penicillium*, and *Cladosporium* were exclusively isolated from Hunan province, while *Paraboeremia*, *Podospora*, *Phoma*, and *Xylariales* were unique to Hubei province. In addition, some endophytic fungi could not be identified due to the absence of sporulation on PDA (Potato Dextrose Agar) medium. Overall, the endophytic fungi from Hunan province exhibited higher diversity but lower abundance compared to those from Hubei province.

### 2.5. Screening of HupA-Producing Endophytic Fungi

#### 2.5.1. Detection of Alkaloid Precipitator

To rapidly identify alkali-producing endophytic fungi, the purified fungal isolates were cultivated in shake flasks. Following a 7-day incubation period, 3 mL aliquots of the fermentation supernatant were individually mixed with three drops of potassium mercuric iodide, bismuth potassium iodide, and silicotungstic acid [9]. Fungal samples that exhibited precipitation were further processed by scale-up fermentation, acid–base extraction, and subsequent HPLC and LC-MS analyses.

#### 2.5.2. HPLC and LC-MS Analysis

**Figure 3:**
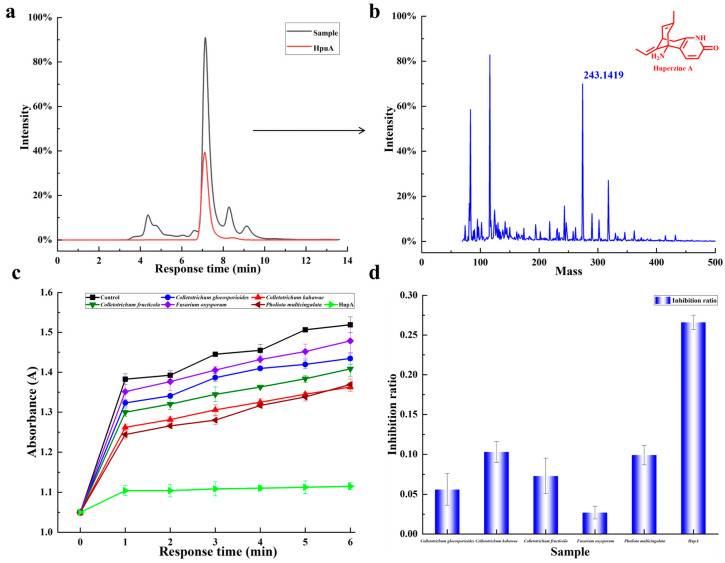
LC-MS analysis of HupA extracted from the mycelia of endophytic fungi and its inhibitory effect against AChE. (**a**,**b**). LC-MS analysis of HupA ([M + H]⁺ = *m*/*z* 243.1419) produced by endophytic fungi. (**c**,**d**). Absorbance of AChE and the inhibitory activity of HupA towards AChE, with HupA extracted from four endophytic fungi: *C. gloeosporioides*, *C. kahawae*, *C. fructicola*, and *F. oxysporum*. The arrow indicates the mass spectral signal corresponding to the peak. Data are presented as the mean ± standard deviation of three biological replicates (*n* = 3).

For HPLC analysis, standard HupA was used as the control to confirm the presence of HupA. A characteristic absorption peak at 310 nm was observed for alkaloids extracted from the mycelia of the following four endophytic fungi: *Colletotrichum gloeosporioides*, *C. kahawae*, *Colletotrichum fructicola*, and *Fusarium oxysporum* (Figure 3a). LC-MS analysis was further conducted to validate these findings. As shown in Figure 3b, both the standard HupA and the fungal samples exhibited a similar molecular weight ([M + H]⁺ = *m*/*z* 243.1419), confirming that these four fungi could synthesize HupA. Among them, *C. kahawae* produced the highest concentration of HupA, reaching 37.94 μg/g (dry cell weight, dcw). Therefore, *C. kahawae* was chosen as the optimal fermentation strain to overproduce HupA.

### 2.6. Inhibition Activity of Purified HupA on AChE

Given that the four endophytic fungi were capable of producing HupA, we subsequently investigated whether the extracted HupA could inhibit AChE. Encouragingly, the HupA derived from the fermentation broth demonstrated time-dependent inhibitory activity against AChE (Figure 3c). However, this activity was lower than that of the standard HupA (Figure 3d), likely due to the crude nature of the extracted HupA.

### 2.7. Strategies for Improving HupA Production in Endophytic Fungi

#### 2.7.1. Selective Medium

**Figure 4:**
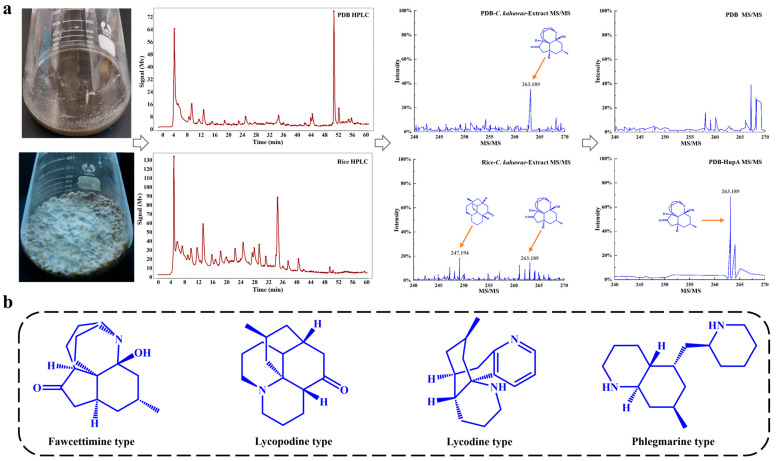
Effects of PBD medium and rice medium on HupA biosynthesis. (**a**). *C. kahawae* was cultured in PDB and rice medium, with secondary metabolites analyzed using HPLC and MS/MS to identify differences. (**b**). Parent nucleus of four types of Lycopodium alkaloids from *H. serrata*.

In this study, the endophytic fungus *C. kahawae* was cultivated independently in PDB (Potato Dextrose Broth) liquid medium and rice solid medium. LC-MS analysis revealed that *C. kahawae* produced a higher level of HupA in PDB medium compared to rice medium (Figure 4a). Given that the Lycopodium alkaloids produced by *H. serrata* are structurally categorized into four groups—fawcettimine, lycopodine, lycodine, and phlegmarine [21] (Figure 4b)—we hypothesized that *C. kahawae* might also synthesize these compounds. As anticipated, *C. kahawae* was capable of biosynthesizing Lycopodium alkaloids, which were successfully detected in the fermentation broth (Figure 4). MS/MS analysis showed that the fragmentation peak signal for fawcettimine was stronger in PDB medium than in rice medium. However, additional fragmentary peaks for fawcettimine and lycopodine were observed in rice medium, albeit with weaker signals (Figure 4a). Collectively, these results suggest that PDB medium is more conducive to the production of Lycopodium alkaloids, such as HupA, while rice medium may enhance the diversity of metabolites produced. Considering our research objectives, we selected PDB medium as the basic medium for subsequent experiments.

#### 2.7.2. Effects of Biological Precursor and Inducers on HupA Production

**Figure 5:**
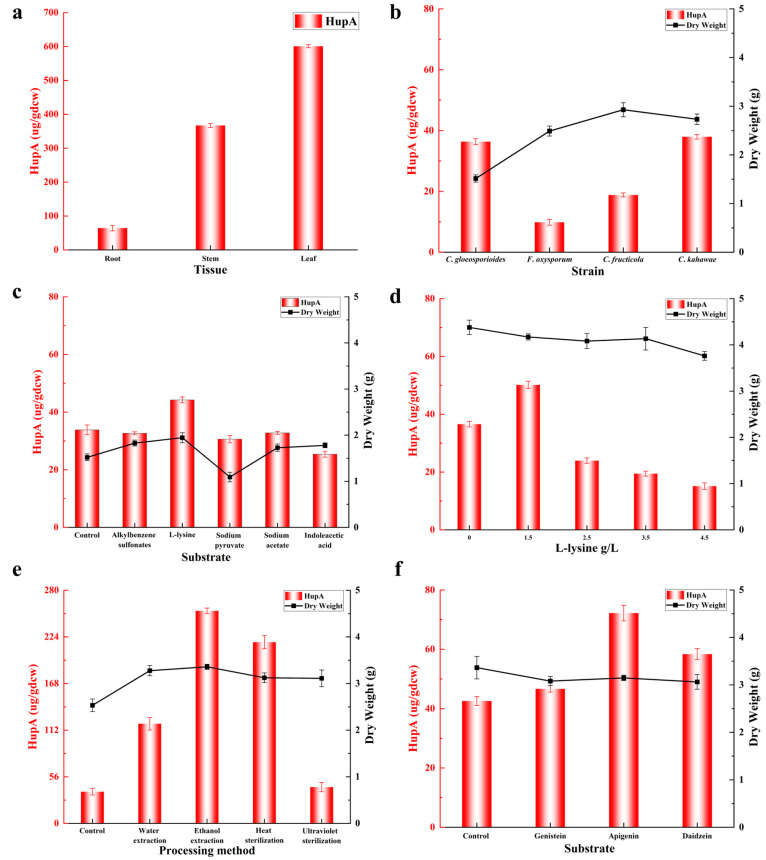
Levels of HupA in *H. serrata* tissues and its endophytic fungi. (**a**). Levels of HupA in the root, stem, and leaf of *H. serrata*. (**b**). Levels of HupA in the mycelia of four endophytic fungi. (**c**–**f**). The effects of adding fermentation ingredients on HupA production in *C. kahawae*. (**c**). L-lysine as a precursor and biological inducers (alkylbenzene sulfonates, sodium pyruvate, sodium acetate, and indoleacetic acid). (**d**). Different concentrations of L-lysine. (**e**). Various processing methods of *H. serrata*, such as extraction methods (water extraction and ethanol extraction) and sterilization methods (heat sterilization and ultraviolet sterilization). (**f**). Flavonoids, namely genistein, apigenin, and daidzein. The four endophytic fungi refer to *C. gloeosporioides*, *C. kahawae*, *C. fructicola*, and *F. oxysporum*. The HupA content is expressed as HupA per gram of mycelium dry cell weight (dcw). The data presented are the mean ± standard deviation (s.d.) from biological triplicates (*n* = 3).

Recent studies have highlighted the significance of fermentation optimization and the use of inducers in enhancing the production of economically valuable chemicals from endophytic fungi [22]. In the current study, we investigated the effects of L-lysine and various inducers (alkylbenzene sulfonates, sodium pyruvate, sodium acetate, and indoleacetic acid) on HupA production by *C. kahawae* by adding them to the fermentation broth at a final concentration of 0.5 g/L. In vitro experiments revealed that the growth of *C. kahawae* was significantly promoted by L-lysine, alkylbenzene sulfonates, sodium acetate, and indoleacetic acid. Interestingly, sodium pyruvate at the same concentration slightly inhibited fungal growth (Figure 5c). Regarding HupA production, alkylbenzene sulfonates, sodium acetate, and indoleacetic acid led to a modest decrease in HupA formation compared to the control. In contrast, L-lysine supplementation resulted in a substantial increase in HupA yield, reaching 44.22 μg/g dcw, which represents a 30.66% improvement over the control (Figure 5c).

#### 2.7.3. Optimization of L-Lysine Concentration for Improving HupA Production

Given that 0.5 g/L L-lysine could enhance HupA production, we further optimized its concentration. To this end, *C. kahawae* was cultivated in shake flasks supplemented with L-lysine at concentrations of 1.5, 2.5, 3.5, and 4.5 g/L. A control flask without L-lysine supplementation was also prepared. Interestingly, fungal growth remained largely unchanged across different L-lysine concentrations compared to the control (Figure 5d). However, 4.5 g/L L-lysine slightly inhibited *C. kahawae* growth. HPLC analysis showed that supplementing with 1.5 g/L L-lysine resulted in a 40% increase in HupA production, reaching 50.17 μg/g dcw, compared to the control. In contrast, higher concentrations of L-lysine (2.5, 3.5, and 4.5 g/L) led to decreased HupA production (Figure 5d). These results indicate that while L-lysine supplementation can significantly boost HupA yield, excessive L-lysine not only stagnates cell growth but also impairs HupA formation. Therefore, optimal L-lysine concentration is crucial for maximizing HupA production.

#### 2.7.4. Effects of *H. serrata* Extracts on HupA Production

*H. serrata* contains a variety of essential components for HupA synthesis, including cofactors, intermediates, and substrates [10]. Given that processing methods may influence HupA production, we examined the effects of different extraction methods (water extraction and ethanol extraction) and sterilization methods (heat sterilization and ultraviolet sterilization) on HupA yield. Briefly, four groups of fresh *H. serrata* leaves of uniform quality were crushed and subjected to water extraction, ethanol extraction, heat sterilization, or ultraviolet sterilization before being added to the shake-flask culture medium of *C. kahawae*. Results indicated that different solvents (water or ethanol) had minimal effects on *C. kahawae* growth and HupA yield in control cultures, whereas the addition of *H. serrata* extracts significantly enhanced the growth of *C. kahawae*. Moreover, both sterilization and extraction methods were crucial factors influencing HupA production. As shown in Figure 5e, after normalization to endogenous HupA levels in *H. serrata* leaves, ultraviolet sterilization and water extraction increased HupA production by 14.65% (43.61 μg/g dcw) and 214.62% (119.68 μg/g dcw), respectively, compared to the control. In contrast, heat sterilization and ethanol extraction led to a remarkable increase in HupA yield by 472.38% (217.73 μg/g dcw) and 571.20% (255.32 μg/g dcw), respectively. In conclusion, the presence of essential precursors and cofactors in *H. serrata* extracts promoted HupA biosynthesis. Additionally, the choice of extraction method was critical for optimizing HupA yield.

#### 2.7.5. Effects of Flavonoids on HupA Production

*H. serrata* is rich in alkaloids, triterpenoids, and flavonoids, with flavonoids serving as highly specific chemical signals that play a crucial role in plant–endophyte interactions [23]. Additionally, flavonoids can stimulate microbial metabolism, primarily due to their acidic protons and redox activity [24]. To investigate their potential impacts on HupA production, three flavonoids—genistein, apigenin, and daidzein (each at 0.5 g/L)—were directly added to the fermentation medium. While these flavonoids had minimal effects on the growth of *C. kahawae*, they significantly influenced HupA production (Figure 5f). Specifically, genistein resulted in a modest 9.61% increase in HupA yield (46.66 μg/g dcw). In contrast, daidzein and apigenin led to more substantial increases of 37.13% (58.38 μg/g dcw) and 69.62% (72.21 μg/g dcw), respectively. Clearly, among the tested flavonoids, apigenin was the most effective in enhancing HupA biosynthesis, outperforming both genistein and daidzein. In summary, flavonoids mitigated oxidative stress in *C. kahawae* [25], thereby creating a favorable environment for HupA biosynthesis.

### 2.8. Transcriptome Sequencing of H. serrata

**Figure 6:**
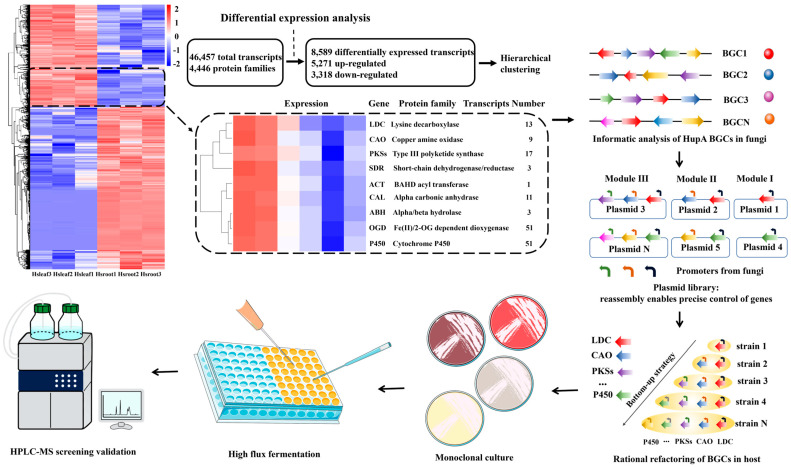
Proposed biofoundry workflow for genetic modification to elucidate the HupA biosynthetic pathway, based on upregulated transcripts of associated genes in *H. serrata*. The workflow comprises several modules: transcriptomic-guided identification of candidate HupA biosynthetic genes from *H. serrata*, bioinformatic analysis of biosynthetic gene clusters (BGCs), automated assembly for plasmid library construction, rational refactoring of BGCs in strains, monoclonal culture, high flux fermentation, and LC-MS screening validation. The arrows indicate the direction or sequence of the process. The ellipsis (…) indicates the omission of genes including SDR, ACT, CAL, ABH, and OGD.

**Figure 7:**
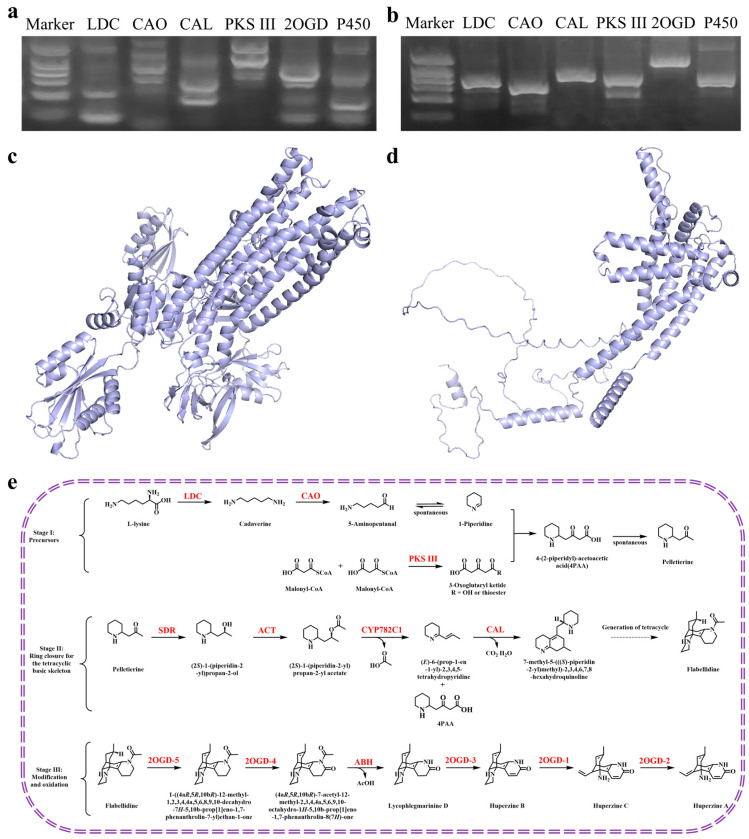
Effects of *H. serrata*-induced and non-induced *C. kahawae* on the expression of enzymes involved in HupA biosynthesis. (**a**). Colony PCR of non-induced *C. kahawae*, which showcases complex and diverse electrophoretic bands. (**b**). Colony PCR of *H. serrata* extract-induced *C. kahawae*, showing clear and strong electrophoretic bands. (**c**). The tertiary structure of PKS III derived from the PCR cloning of *H. serrata*-induced *C. kahawae*. (**d**). The tertiary structure of 2OGD derived from the PCR cloning of *H. serrata*-induced *C. kahawae*. (**e**). The proposed biosynthetic pathway of HupA. Abbreviations: LDC, lysine decarboxylase; CAO, copper amine oxidase; CAL, alpha carbonic anhydrase; PKS III, type III polyketide synthase; 2OGD, Fe (II)/2-OG dependent dioxygenase; P450, cytochromes P450.

In this study, extraction experiments demonstrated that the new leaves of *H. serrata* contain higher levels of HupA compared to its roots (Figure 5a). This finding provides valuable guidance for extracting genomic DNA or RNA to elucidate the HupA biosynthetic pathway. To construct a comprehensive transcriptomic dataset of *H. serrata* from Hubei province, both leaves and roots were subjected to long-read and short-read RNA sequencing (RNA-seq). Based on the transcriptomic results, differentially expressed genes were identified, and hierarchical clustering was performed to generate co-expressed clusters of transcripts (Figure 6). This analysis revealed a total of 46,457 transcripts and 4446 protein families. Among these, 5271 transcripts were upregulated and contained key biosynthetic enzymes previously reported (Figure 6 and Figure 7e), including lysine decarboxylase (LDC), copper amine oxidase (CAO), type III polyketide synthase (PKS III), and Fe(II)/2-oxoglutarate-dependent dioxygenase (2OGDs) [6]. This cluster was highly enriched with transcripts encoding metabolic enzymes involved in the biosynthesis of natural products, such as cytochromes P450 (CYPs), 2OGDs, short-chain dehydrogenase/reductase (SDR), BAHD acyltransferase (ACT), alpha-carbonic anhydrase (CAL), and alpha/beta hydrolase (ABH). These enzymes collectively suggest the presence of a machinery capable of HupA scaffold biosynthesis [8]. These data provide compelling evidence to support the subsequent co-expression of key genes in endophytic fungi.

### 2.9. H. serrata Extracts Upregulated the HupA Biosynthesis Genes in C. kahawae

The addition of *H. serrata* extracts to PDB medium significantly enhanced HupA production in *C. kahawae* compared to the control (Figure 5e). A comprehensive analysis of the *H. serrata* transcriptome revealed numerous upregulated genes, some of which are associated with HupA biosynthesis (Figure 7e). Based on these findings, we designed specific primers (Table S3) to clone genes from both *H. serrata*-induced and non-induced *C. kahawae* genomes to investigate whether horizontal gene transfer occurred between *C. kahawae* and *H. serrata*. As shown in Figure 7a,b, the electrophoretic bands in the treatment group were clear and strong; whereas, those in the control group were complex and diverse. This suggests that the low HupA production in *C. kahawae* was likely attributed to the silence or weak expression of related genes; whereas, the addition of *H. serrata* extracts upregulated the expression of genes involved in HupA biosynthesis in *C. kahawae*. PCR cloning and sequencing of the prominent bands from *C. kahawae* revealed low homology with both *H. serrata* transcripts and the reported enzyme genes involved in HupA biosynthesis. Subsequent homology modeling was conducted on the amino acid sequences obtained from these prominent bands, revealing that only PKS III and 2OGD could form tertiary structures (Figure 7c,d). In short, this low sequence similarity likely indicates genetic independence between *H. serrata* and *C. kahawae*, suggesting that the enhanced HupA production in *C. kahawae* may be due to regulatory effects rather than horizontal gene transfer.

## 3. Discussion

The variation in HupA content in *H. serrata* plants across different provinces may be attributed to a combination of factors, including geographical environment, climate conditions, genetic variation, collection time, and extraction methods. In this study, we first investigated *H. serrata* plants from various regions in China to identify those with the highest potential for HupA biosynthesis. Next, we focused on the endophytic fungi from *H. serrata* collected in Hunan and Hubei provinces (Figure 2a,b), as four of these fungi were able to independently synthesize HupA. Among them, *C. kahawae* showed the highest HupA production and was thus selected for fermentation optimization using the OSMAC strategy. We found that the precise addition of substrates or intermediate metabolites to the fermentation medium remarkably improved HupA production. While the addition of L-lysine and *H. serrata* extracts to the fermentation medium of *C. kahawae* led to HupA yields of 50.17 µg/g dcw and 255.32 µg/g dcw, respectively (Figure 5d,e), the inclusion of *H. serrata* flavonoids daidzein and apigenin in the fermentation medium led to 58.38 µg/g dcw and 72.21 µg/g dcw of HupA, respectively (Figure 5f). Clearly, *H. serrata* extract was most effective in enhancing HupA production, as it contains all the necessary nutrients for *C. kahawae* growth. Subsequently, we utilized specific primers to clone parts of the HupA biosynthesis genes (*LDC*, *CAO*, *CAL*, *PKS III*, *2OGD*, *P450*) from both *H. serrata* and *H. serrata*-induced *C. kahawae*. Surprisingly, the HupA biosynthesis genes from *C. kahawae* demonstrated low sequence similarity with those from *H. serrata*, indicating relative independence between *H. serrata* and *C. kahawae* in HupA biosynthesis.

The initial strain is crucial for the high-level production of desired metabolites. Given that some endophytes can independently synthesize HupA after long-term coexistence with the host plant *H. serrata* [26], we systematically investigated the endophytes of *H. serrata* to identify the best-performing strain for HupA production. Since *H. serrata* from Hunan and Hubei provinces exhibited higher levels of HupA (Figure 1), and the leaves contained more HupA than other tissues (Figure 5a), we isolated endophytic fungi from the fresh leaves of *H. serrata* and subsequently identified them based on morphologies and ITS sequences. Notably, *Colletotrichum* spp. were predominant in both regions in terms of HupA production (Tables S1 and S2). Among the diverse isolates, four endophytic fungi, including *C. gloeosporioides*, *C. kahawae*, *C. fructicola*, and *F. oxysporum*, were capable of producing HupA, as confirmed by alkaloid precipitation, HPLC, and LC-MS analyses (Figure 5b). Although the yield of HupA was low, it demonstrated the reversible inhibition of AChE (Figure 3c,d and Figure 5b).

The OSMAC strategy is a viable approach to upregulate low-expression genes or even activate silent genes. In this study, we optimized fermentation conditions to enhance HupA production by *C. kahawae*. Specifically, the fermentation medium was supplemented with the L-lysine, biological inducers (such as alkylbenzene sulfonates, sodium pyruvate, sodium acetate, and indoleacetic acid), and somatomedin (derived from *H. serrata* extracts). Consequently, the addition of L-lysine and *H. serrata* extracts significantly promoted both cell growth and HupA synthesis (Figure 5c–e). This effect may be attributed to L-lysine serving as an initial substrate and *H. serrata* extracts providing nearly all the metabolites and, potentially, enzymes necessary for HupA biosynthesis. In addition to L-lysine and *H. serrata* extracts, flavonoids—including genistein, apigenin, and daidzein—were independently added to the fermentation medium to boost HupA production. Similarly, daidzein and apigenin notably enhanced HupA yields (Figure 5f). Transcriptomic analysis revealed that the supplementation of *H. serrata* extracts significantly upregulated several HupA biosynthesis genes in *C. kahawae* (Figure 7a,b,e).

While optimizing fermentation conditions can enhance HupA production by upregulating low-expressed or even silent genes, the full potential of endophytic fungi for HupA synthesis remains untapped. This limitation arises from the incomplete identification of HupA biosynthesis genes, which hampers comprehensive efforts to identify and eliminate metabolic bottlenecks. Fortunately, significant advancements in “meta-omic” technologies increase the likelihood of decoding the genomes and regulatory networks of both *H. serrata* and its endophytes [27,28]. Indeed, the transcriptomic data generated in this study have identified a series of differentially expressed genes involved in HupA biosynthesis (Figure 6 and Figure 7e), corroborating previous findings [6,8]. Given that comparative genomics can identify homologous genes, we propose that integrating sequence alignment, hierarchical clustering (Figure 6), machine learning, and in vitro experimental validation will enable the complete elucidation of the HupA biosynthesis pathway. We also envision that the isolated endophytic fungi, once subjected to genome editing and global regulatory optimization, will serve as ideal hosts for overproducing HupA.

## 4. Materials and Methods

### 4.1. Plant Materials

In China, *H. serrata* is primarily growing in the Yangtze River basin and southern China, with sporadic occurrences in the northeastern areas [29]. To identify the level of HupA, *H. serrata* samples were collected from moist forests and rock crevices at elevations ranging from 300 m to 2700 m. These samples were sourced from various locations, including Huaihua City in Hunan Province, Chihe City in the Guangxi Zhuang Autonomous Region, Bozhou City in Anhui Province, Kunming City in Yunnan Province, Enshi City in Hubei Province, and Jilin City in Jilin Province.

### 4.2. Fingerprint Analysis

The *H. serrata* samples collected from the six aforementioned regions in China were cleaned and dried at 35 °C to preserve thermolabile bioactive compounds. The 250 g samples were then finely ground into powder and dissolved in ethanol to extract the bioactive ingredients [30]. Over the 40-day extraction period, intermittent agitation was performed, and samples were taken every 10 days to analyze the components. After thorough filtration, the filtrate and the occluded solution in the solid residue were combined and concentrated under reduced pressure to obtain a crude extract for subsequent HPLC analysis.

### 4.3. Isolation of Endophytic Fungi

Endophytic fungi are typically isolated from plant tissues following rigorous surface disinfection, and their endophytic nature is confirmed by directly amplifying DNA from the tissues. In this study, endophytic fungi were isolated from the healthy leaves of *H. serrata* using a previously established method [9,20,31]. Briefly, fresh *H. serrata* leaves were first cleaned with tap water and then sterilized in 75% ethanol for 3 min. The leaves were subsequently rinsed three times with sterile distilled water and dried on sterile filter paper. The leaves were then cut into small pieces using a sterile scalpel and inoculated onto PDA plates containing antibiotics to thwart bacterial growth. A PDA plate without any inoculum served as the control. The plates were incubated at 28 °C to observe the emergence and growth of endophytic fungi from the leaf fragments. Pure fungal isolates were obtained by repeatedly transferring and incubating individual hyphal tips onto fresh PDA medium [20]. Next, single-spore isolation was conducted. The final pure cultures were numbered and transferred to PDA slant tubes for storage at 4 °C. Alternatively, spores and mycelia could also be preserved in 15% glycerol at −20 °C. The majority of the isolated endophytic fungi were deposited at Beijing University of Chemical Technology.

### 4.4. Identification of Endophytic Fungi

To investigate the diversity of endophytic fungi in *H. serrata* samples from Hunan and Hubei provinces, a combination of conventional culture methods and high-throughput sequencing was employed. The identification of endophytic fungi was based on the morphology of conidia and colonies, as well as unique phenotypic characteristics [20]. Additionally, the ITS region of the endophytic fungi was amplified via PCR. Genomic DNA was extracted using the Quick-gDNA™ Mini Prep kit from Zymo Research (Irvine, CA, USA). The ITS region was then amplified and sequenced by Beijing Ruibo Xingke Biotechnology Co., Ltd. (Beijing, China), using the universal primers ITS1 (5′-TCCGTAGGTGAACCTGCGG-3′) and ITS4 (5′-TCCTCCGCTTATTGATATGC-3′) [20]. The resulting ITS sequences were subjected to BLAST searches against the GenBank database to identify homologous sequences for genus- or species-level classification. Phylogenetic analysis was performed using MEGA version 7.0 [32], and the phylogenetic tree was constructed using the neighbor-joining method and visualized with the iTOL program.

### 4.5. Shake Flask Fermentation of Endophytic Fungi

The OSMAC strategy is a powerful approach tailored to activate silent gene clusters and enhance the production of diverse metabolites by altering medium compositions, culture conditions, and employing co-cultivation techniques [12,13,14,15,16,17]. Inspired by this strategy, endophytic fungi from *H. serrata* were cultured in both PDB liquid medium and rice solid medium. Initially, the isolated endophytic fungi were cultured on PDA plates for 3–5 days. Spores were then rinsed from the plates with sterile water, and the spore concentration was adjusted to 10^6^/mL using a hemocytometer under an optical microscope. The spore suspension was individually inoculated into the culture medium at a 1% (*v*/*v*) ratio, followed by incubation at 28 °C and 180 r/min for 7–10 days in PDB or 35–40 days in rice medium. For preliminary screening, endophytic fungi were cultured in 250 mL shake flasks, each containing 100 mL of PDB, with three replicates per treatment. For large-scale cultivation, endophytic fungi were incubated in 1 L shake flasks, each containing 250 mL of PDB, again with three replicates per treatment. Additionally, the culture medium was supplemented with various components, including precursors (e.g., L-lysine), biological inducers (e.g., alkylbenzene sulfonates, sodium pyruvate, sodium acetate, and indoleacetic acid), flavonoid intermediates (e.g., genistein, apigenin, and daidzein), and somatomedin (present in plant extracts).

### 4.6. H. serrata and Its Extracts

Fresh *H. serrata* leaves were rinsed with deionized water, dried at 35 °C, and pulverized. For sterilization, 0.2 g of powder was treated with either 30 min heat sterilization at 115 °C or 3 h ultraviolet sterilization (254 nm, 20 W, 30 cm working distance). The sterilized powder was then aseptically incorporated into the medium, homogenized, and dispensed into shake flasks. For extraction, 0.2 g of *H. serrata* powder was combined with 20 mL of ethanol or deionized water, followed by ultrasound-assisted extraction at 30 °C for 3 h. After filtration through 0.22 μm membrane filters, the ethanol extracts were concentrated to 6 mL (0.033 g/mL) under vacuum, while the water extracts were lyophilized and redissolved in 6 mL of water. Aliquots (3 × 2 mL) of each extract were prepared and sterilized for subsequent fermentation inoculation. Parallel solvent controls (water vs. ethanol) were included to assess solvent effects.

### 4.7. HupA Extraction from Endophytic Fungi

Based on the chemical properties of HupA and previous studies [33,34], HupA was extracted from PDB-cultured mycelia using an acid–base method [35]. Additionally, the fermentation leachate from rice medium cultures was collected, and HupA was isolated via ethyl acetate gradient extraction. The extracted HupA was then purified and concentrated under reduced pressure using a rotary evaporator.

### 4.8. Analytical Method

#### 4.8.1. Alkaloid Precipitator

HupA is an unsaturated sesquiterpene alkaloid containing a pyridone moiety and a primary amino group [34]. Alkaloids can be detected using Dragendorff’s reagent [36,37], which reacts with the positively charged groups (e.g., amino groups) of alkaloids to form water-insoluble salts. Commonly used alkaloid precipitators include potassium mercuric iodide, bismuth potassium iodide, and silicotungstic acid [9]. Upon reaction with alkaloids, these reagents lead to yellow, yellow-brown, and gray-white precipitates, respectively. Thus, endophytic fungi capable of producing alkaloids can be readily identified through this method [36,37].

#### 4.8.2. HPLC and LC-MS Analysis of HupA

To accurately quantify metabolites, the crude extract was obtained by centrifugation at 12,000 rpm for 10 min. The resulting supernatant was filtered through a 0.22 μm membrane and stored at 4 °C. Metabolite analysis was performed using an HPLC system (Shimadzu, Kyoto, Japan) equipped with a C18 column and an SPD-20A UV detector set at 310 nm. The column temperature was maintained at 30 °C, and the mobile phase consisted of methanol and water (containing 0.1% formic acid) in a ratio of 70:30 (*v*/*v*). The flow rate was set at 0.6 mL/min. HupA was quantified using a standard curve generated with a reference standard from Shanghai Bide Pharmaceutical Technology Co., Ltd. (Shanghai, China), with concentrations ranging from 0 to 100 mg/L. The absorbance peak areas and HupA concentrations exhibited a linear relationship (R^2^ = 0.9994). The presence of HupA in the endophytic fungal extracts was further confirmed by LC-MS. The electrospray ionization mass spectrometry (ESI-MS) spectrum was acquired using a Waters system. A sample volume of 4 µL was injected into a Waters (Milford, MA, USA) ACQUITY UPLC system with a BEH C8 column (1.7 µm, 2.1 × 100 mm). The gradient elution was performed using solvent A (water + 0.1% formic acid) and solvent B (acetonitrile) for 15 min at a flow rate of 0.3 mL/min.

### 4.9. In Vitro Assay of AChE Activity

The inhibitory activity of HupA extract against AChE was evaluated using Ellman’s method [9,20]. Briefly, a mixture of 125 µL of 0.1 M phosphate buffer (pH 8.0), 50 µL of 0.4 U/mL AChE, 25 µL of 7.6 mM dithiobis nitrobenzoic acid (DTNB), and 20 µL of HupA extract was prepared in a 96-well plate. The mixture was incubated at 30 °C for 30 min, followed by the addition of 30 µL of 6.2 mM acetylthiocholine iodide (ATCI). The absorbance of the mixture was then measured at 412 nm using a microplate reader, with readings taken every minute for 6 min. AChE activity was normalized to the control measurements. All assays were performed in triplicate, and the percentage inhibition was calculated using the following formula: I% = (Absorbance of control − Absorbance of sample)/Absorbance of control [20,38].

## 5. Conclusions

In this study, we systematically investigated the endophytic fungi of *H. serrata* sourced from two provinces in China. Among the isolates, four endophytic fungi were able to produce bioactive HupA. By employing the OSMAC approach, supplementing the fermentation medium of *C. kahawae* with L-lysine, flavonoids, and *H. serrata* extracts led to 40%, 69.62%, and 571.20% increases in the production of HupA, respectively. Of particular significance is that *H. serrata* extracts can upregulate the HupA biosynthesis genes and in turn boost HupA production, because the extracts contain all the necessary nutrients for *C. kahawae* growth. This study underscores a nutritional exchange between *H. serrata* and *C. kahawae*, providing valuable insights for utilizing plant extracts to overproduce the desired metabolites in endophytic fungi.

## Data Availability

The data involved in the research are included in manuscript. All relevant data are available upon reasonable request from the corresponding author.

## Supplementary Materials

The following supporting information can be downloaded at https://www.mdpi.com/article/10.3390/molecules30132704/s1, Table S1. ITS region sequences of 30 endophytic fungi from Huaihua City, Hunan Province. Table S2. ITS region sequences of 58 endophytic fungi from Enshi City, Hubei Province. Table S3. Primers used in this study.
