# Supplementary material for: Systematic Investigations of the Huperzine A—Producing Endophytic Fungi of Huperzia serrata in China and Fermentation Optimization Using OSMAC Strategy
Source: Molecules. 2025 Jun 23;30(13):2704. doi: 10.3390/molecules30132704 (PMC12251189; doi:10.3390/molecules30132704)
Supplement: Supplementary file 1 [file molecules-30-02704-s001.zip › molecules-3560319-supplementary.pdf]

< Supplementary Material>

Systematic Investigations of the Huperzine A-Producing Endophytic Fungi of *Huperzia serrata* in China and Fermentation Optimization Using OSMAC Strategy

Wei Li, Zhicheng Wang, Qiuyu Zhu, Pingfang Tian*

Beijing Key Laboratory of Bioprocess, College of Life Science and Technology, Beijing University of Chemical Technology, Beijing 100029, P. R. China

Wei Li E-mail: 2021400299@buct.edu.cn;

Zhicheng Wang E-mail: 2023201234@buct.edu.cn;

Qiuyu Zhu E-mail: 2022400332@buct.edu.cn;

Corresponding Author

Pingfang Tian E-mail: tianpf@mail.buct.edu.cn.

Table S1 ITS region sequences of 30 endophytic fungi from Huaihua City, Hunan Province

Number	Scientific Name	GenBank	Per Identity (%)
1	*Colletotrichum fructicola*	OQ652420.1	99.81%
	Colletotrichum fructicola	OQ652405.1	99.81%
2	*Colletotrichum gloeosporioides*	FJ459940.1	100.00%
	Colletotrichum fructicola	OQ652288.1	99.81%
3	*Colletotrichum fructicola*	OQ652288.1	100.00%
	Colletotrichum fructicola	OQ927180.1	100.00%
4	*Colletotrichum fructicola*	OQ652417.1	99.81%
	Colletotrichum fructicola	OQ652394.1	99.81%
5	*Whalleya microplaca*	MN844248.1	99.30%
	Whalleya microplaca	MW497232.1	99.64%
6	*Whalleya microplaca*	MN844248.1	98.59%
	Whalleya microplaca	MT138724.1	98.92%
7	*Whalleya microplaca*	MN844248.1	99.65%
	Whalleya microplaca	MT138724.1	100.00%
8	*Whalleya* sp.	KX722233.1	99.64%
	Whalleya microplaca	MW497232.1	99.64%
9	*Whalleya microplaca*	MN844248.1	99.47%
	Whalleya microplaca	MN844247.1	99.82%
10	*Whalleya* sp.	KX722233.1	99.82%
	Whalleya microplaca	MN844248.1	99.82%
11	*Annulohypoxylon stygium*	MF770833.1	99.77%
	Fungal endophyte	KY022823.1	99.77%
12	*Xylariaceae* sp.	MK611677.1	99.43%
	Xylariaceae sp. JL8	KM513576.1	99.32%
13	*Colletotrichum siamense*	MW228101.1	99.81%
	Colletotrichum sp.	MT577044.1	100.00%
14	*Pestalotiopsis microspora*	MN272355.1	100.00%
	Colletotrichum gloeosporioides	MZ648282.1	99.82%
15	*Phyllosticta capitalensis*	KX262976.1	100.00%
	Fungal sp.	MG779574.1	100.00%
16	*Phyllosticta* sp. FL2-PL1	KP900240.1	99.49%
	Phyllosticta capitalensis	OQ847683.1	99.49%
17	*Fungal* sp.	MG779574.1	99.83%
	Fungal endophyte	KF435675.1	99.83%
18	*Phyllosticta capitalensis*	MH865128.1	99.67%
	Phyllosticta capitalensis	MH880250.1	99.67%
19	*Phyllosticta capitalensi*	KP900296.1	99.43%
	Colletotrichum truncatum	MN429225.1	99.62%
20	*Colletotrichum truncatum*	MG004780.1	99.62%
	Colletotrichum sp. 1 RJ-2019	MZ078540.1	99.62%
	Hypoxylon sp. LS11	KM520034.1	98.43%

21	*Hypoxylon* sp.	MG198894.1	97.92%
22	*Nigrospora sphaerica*	OR083393.1	99.61%
	Nigrospora sphaerica	KU360636.1	99.61%
23	*Nodulisporium* sp.	LC504946.1	99.89%
	Sordariomycetes sp.	MT183794.1	99.78%
24	*Colletotrichum acutatum*	FJ478064.1	99.82%
	Colletotrichum fioriniae	MT068552.1	99.63%
25	*Colletotrichum fioriniae*	MN429169.1	99.82%
	Colletotrichum fioriniae	OR029560.1	99.82%
26	*Daldinia childiae*	MZ573043.1	99.44%
	Daldinia childiae	MN844359.1	99.08%
27	*Penicillium paxilli*	MN511334.1	99.26%
	Penicillium paxilli	JN617687.1	99.26%
28	*Cladosporium* sp.	MG975642.1	100.00%
	Cladosporium sp.	MG975639.1	100.00%
29	*Fusarium oxysporum*	MT732036.1	100.00%
	Phomopsis sp.	MZ855439.1	99.45%
30	*Colletotrichum* sp.	MT577041.1	100.00%
	Colletotrichum fructicola	MT211270.1	100.00%

Table S2 ITS region sequences of 58 endophytic fungi from Enshi City, Hubei Province

Number	Scientific Name	Genbank	Per Identity (%)
1	*Colletotrichum gloeosporioides*	MT568599.1	99.64%
	Colletotrichum gloeosporioides	MT568594.1	99.65%
2	*Podospora* sp.	MK429842.1	100.00%
	Paraboeremia sp.	MH230278.1	100.00%
3	*Phyllosticta capitalensis*	MT085755.1	99.83%
	Phyllosticta capitalensis	MT071244.1	99.66%
4	*Colletotrichum higginsianum*	MN429284.1	99.81%
	Colletotrichum higginsianum	MN429283.1	99.81%
5	*Colletotrichum truncatum*	KP689256.1	99.81%
	Phyllosticta capitalensis	KP900296.1	99.62%
6	*Phyllosticta capitalensis*	OQ847682.1	99.83%
	Phyllosticta capitalensis	MF595520.1	99.66%
7	*Nigrospora sphaerica*	MN153452.1	99.81%
	Nigrospora sphaerica	LC514689.1	99.61%
8	*Colletotrichum boninense*	MN856348.1	99.82%
	Colletotrichum boninense	OQ596451.1	99.82%
9	*Colletotrichum siamense*	MW653779.1	99.45%
	Colletotrichum siamense	OQ652502.1	99.45%
10	*Colletotrichum siamense*	MW513772.1	99.45%
	Colletotrichum siamense	MW513753.1	99.45%

11	*Sordariomycetes* sp.	MT183812.1	99.63%
	Colletotrichum siamense	OQ652478.1	99.44%
12	*Colletotrichum gloeosporioides*	KP689204.1	99.81%
	Colletotrichum gloeosporioides	KP900222.1	99.81%
13	*Nigrospora oryzae*	KF998977.1	99.25%
	Nigrospora oryzae	MT177215.1	99.24%
14	*Xylariales* sp.	KX722242.1	100.00%
	Xylariales sp. JS18	KM513623.1	100.00%
15	*Colletotrichum boninense*	ON332141.1	100.00%
	Colletotrichum boninense	MN856348.1	99.63%
16	*Colletotrichum siamense*	OQ652478.1	99.44%
	Colletotrichum siamense	OQ652502.1	99.25%
17	*Colletotrichum boninense*	MN429163.1	99.29%
	Colletotrichum boninense	MN542218.1	99.29%
18	*Colletotrichum siamense*	MW647812.1	99.45%
	Colletotrichum siamense	MN856280.1	99.63%
19	*Colletotrichum boninense*	MN542218.1	99.64%
	Colletotrichum gloeosporioides	MN856361.1	99.46%
20	*Colletotrichum boninense*	KM513575.1	99.46%
	Colletotrichum boninense	MN429163.1	100.00%
21	*Colletotrichum* sp. LWYF83	MT570085.1	99.63%
	Colletotrichum gloeosporioides	KP900293.1	99.45%
22	*Colletotrichum gloeosporioides*	ON238110.1	99.47%
	Colletotrichum gloeosporioides	EU847425.1	99.47%
23	*Colletotrichum gloeosporioides*	KM513611.1	99.63%
	Colletotrichum siamense	MW647832.1	99.45%
24	*Colletotrichum gloeosporioides*	MK673858.1	99.27%
	Colletotrichum siamense	MW513770.1	99.27%
25	*Colletotrichum boninense*	KM513575.1	99.30%
	Colletotrichum boninense	OP466829.1	99.64%
26	*Fungal endophyte*	FJ449913.1	99.82%
	Colletotrichum boninense	KP900269.1	99.82%
27	*Fungal* sp. SK22	KP893223.1	99.47%
	Fungal endophyte	FJ449913.1	99.46%
28	*Colletotrichum gloeosporioides*	EU847425.1	99.82%
	Colletotrichum boninense	MZ312521.1	99.82%
29	*Fungal endophyte*	FJ449913.1	100.00%
	Colletotrichum gloeosporioides	EU847425.1	99.47%
30	*Colletotrichum boninense*	KM520014.1	100.00%
	Fungal endophyte sp. 3134	FJ232906.1	99.64%
31	*Colletotrichum boninense*	KM513575.1	99.65%
	Colletotrichum boninense	OP466829.1	99.64%
32	*Colletotrichum boninense*	JX625165.1	100.00%
	Colletotrichum gloeosporioides	EU847425.1	99.82%

33	*Colletotrichum gloeosporioides*	MN856361.1	99.46%
	Colletotrichum boninense	MN856348.1	99.46%
34	*Colletotrichum gloeosporioides*	KP900288.1	99.45%
	Colletotrichum gloeosporioides	KP900284.1	99.27%
35	*Colletotrichum jiangxiense*	MZ475151.1	99.81%
	Colletotrichum gloeosporioides	KP900257.1	99.27%
36	*Colletotrichum boninense*	KM513575.1	99.29%
	Colletotrichum boninense	OP466829.1	99.64%
37	*Colletotrichum horii*	ON968693.1	99.63%
	Colletotrichum gloeosporioides	KP900293.1	99.82%
38	*Colletotrichum bletillae*	ON332148.1	99.46%
	Colletotrichum liriopes	MG543776.1	99.45%
39	*Colletotrichum boninense*	OP352911.1	99.65%
	Colletotrichum tropicale	OP288226.1	99.65%
40	*Colletotrichum gloeosporioides*	KP900293.1	99.82%
	Colletotrichum gloeosporioides	KM513606.1	99.82%
41	*Colletotrichum liriopes*	OM179491.1	99.44%
	Colletotrichum sp.	MK367553.1	99.44%
42	*Colletotrichum* sp.	MT577041.1	99.27%
	Colletotrichum gloeosporioides	KP900274.1	99.63%
43	*Fungal endophyte*	FJ449913.1	99.82%
	Colletotrichum boninense	KM513575.1	100.00%
44	*Colletotrichum boninense*	MN856348.1	99.30%
	Colletotrichum boninense	OQ596451.1	99.30%
45	*Fusarium tricinctum*	OR150415.1	99.81%
	Uncultured *Fusarium*	MK407264.1	99.62%
46	*Colletotrichum aenigma*	OM663724.1	99.07%
	Colletotrichum gloeosporioides	KP900222.1	98.89%
47	*Xylaria* sp. 5367	JQ862705.1	99.81%
	Fungal sp.	MW603431.1	99.81%
48	*Colletotrichum karsti*	MK934495.1	99.64%
	Fungal sp.	KY040254.1	98.94%
49	*Colletotrichum siamense*	MW513758.1	99.27%
	Colletotrichum siamense	OQ652475.1	99.09%
50	*Colletotrichum* sp. SF3	GU951768.1	99.45%
	Colletotrichum gloeosporioides	KP900274.1	99.27%
51	*Colletotrichum boninense*	MN856352.1	99.47%
	Colletotrichum boninense	OP163584.1	99.29%
52	*Fungal endophyte* sp. 3134	FJ232906.1	99.28%
	Colletotrichum boninense	KM513575.1	99.46%
53	*Colletotrichum gloeosporioides*	ON238110.1	99.47%
	Colletotrichum boninense	KM513575.1	99.12%
54	*Colletotrichum gloeosporioides*	KP900276.1	99.63%
	Colletotrichum gloeosporioides	KP900256.1	99.63%

55	*Colletotrichum boninense*	KM513575.1	99.29%
	Colletotrichum boninense	MN429163.1	99.82%
56	*Phyllosticta capitalensis*	KM513574.1	99.67%
	Phyllosticta capitalensis	MT071244.1	99.67%
57	*Paraboeremia* sp.	MH230278.1	99.57%
	Podospora sp.	MK429842.1	99.57%
58	*Phoma herbarum*	MH059535.1	99.80%
	Phoma herbarum	KP900244.1	99.60%

Table S3 Primers used in this study

Number	Primers	Primer sequences (5'-3')
1	LDC 1-R	AATGATCACCTGGTCTC
	LDC 1-F	TCAATTGCCACCGAT
2	LDC 2-R	CATAGCATTACCAGGTAC
	LDC 2-F	GAAGCAGCTACAACTATA
3	LDC 3-R	TCTGCGTGTTCTGTG
	LDC 3-F	CTGAGACCACGTGATC
4	LDC 4-R	TCTGTGTCTGTATCTGC
	LDC 4-F	TGATGCATATCCGATACC
5	LDC 5-R	GCTATGGAACACTGGAA
	LDC 5-F	GACAATTGGTCCATCTC
6	LDC 6-R	ATGATCACGTGGTCTC
	LDC 6-F	GGTGCAACACCATC
7	LDC 7-R	CATAGATCCATTGCCAG
	LDC 7-F	GCATCAGCATGTCG
8	LDC 8-R	CCTGGTCTCAACTTGG
	LDC 8-F	TGTTGTGGTTCTGAGC
9	LDC 9-R	ATGTAGGGGCATCTG
	LDC 9-F	TTGTAGCAGCTACAACTA
10	LDC 10-R	AATGATCACCTGGTCTC
	LDC 10-F	TCAATTGCCACCGAT
11	LDC 11-R	AGAGAGAAGAGAGGGAG
	LDC 11-F	ACAACTCTTCGAGTGTG
12	LDC 12-R	GTCAGGCTGATGCTT
	LDC 12-F	AATTGCCACCGATGG
13	LDC 13-R	AGGACAGACGGTGGA
	LDC 13-F	TTCAAGCCCTCGTGA
14	LDC 14-R	AATGATCACCTGGTCTC
	LDC 14-F	ATCGTTGTGGTTCTGAG
15	LDC 15-R	CATAGATCCATTGCCAG
	LDC 15-F	GAGACCACGTGATCA
16	LDC 16-R	ACTCATGGGGCTAGTT
	LDC 16-F	TGCAACACCATCATG

17	CAO 1-R	TGTATCCTTCTGCTTGG
	CAO 1-F	CTTCTAGGTGGAGCAG
18	CAO 2-R	GCATACCTGAGGAGGAA
	CAO 2-F	GATCATGGATAACAGCC
19	CAO 3-R	TATCCTTCTGCTTGGTG
	CAO 3-F	ACCTCTGGCCTTTAAG
20	CAO 4-R	TGAATCCCATATCCTTGG
	CAO 4-F	CGGACCACGAGTGTAA
21	CAO 5-R	TGTATCCTTCTGCTTGG
	CAO 5-F	CCAGCCTTCCAGTTGA
22	CAO 6-R	GCCTCCACTCATGAAG
	CAO 6-F	TGAGTGTCTCCATGC
23	CAO 7-R	CCCTTGACAGCATCTG
	CAO 7-F	CTTGACCTTCTCGTCTT
24	CAO 8-R	GCTATAACCAGCGTGA
	CAO 8-F	GTGATGATGCTCTGCA
25	CAO 9-R	TTGTGGCTCTTGCAG
	CAO 9-F	AGGACGTGCATAACC
26	CAO 10-R	AGGACTTGTGCTCCA
	CAO 10-F	TTTACTTCTGCCTCAAG
27	CAO 11-R	TCCTTCTGCTTGGTG
	CAO 11-F	AAGCTCTTCTAGGTGG
28	CAO 12-R	GCTTGCCAATCTCTC
	CAO 12-F	CCACTGATCCAACCAT
29	CAO 13-R	CTCTTCTGAAGCAGAAA
	CAO 13-F	TCTTGCTTGTCTGGG
30	CAO 14-R	CCCATCTCATCTCTGC
	CAO 14-F	CCATAGGATGCCATG
31	CAO 15-R	AGGAAGCTATGAAGAGG
	CAO 15-F	TCCGATAATGAGGATCT
32	CAO 16-R	CCATCCGAGCTTCAT
	CAO 16-F	TGTTGTCAAGGTCATCT
33	CAL 1-R	CGAAGGACTGGAACT
	CAL 1-F	GGCTTCGTGTTCCTC
34	CAL 2-R	CGCAGATGGTTGAGATC
	CAL 2-F	CATCGACTTCGTGATATC
35	CAL 3-R	GGGAAGACTCTATGGC
	CAL 3-F	TTGACTGTGTGCCC
36	CAL 4-R	CAGCCTCACTGAGTTC
	CAL 4-F	GCTGGATTGGTCTGTT
37	CAL 5-R	GGACTACGACTGTGGA
	CAL 5-F	CATCAACACGGTGCT
38	CAL 6-R	TGGGCCATCACACT
	CAL 6-F	GATACTGGAAGTACTGGC

39	CAL 7-R	CCTCACTGCTACTCG
	CAL 7-F	CCGACGTTGAAGACC
40	CAL 8-R	GATCCACATGGTCCAC
	CAL 8-F	CCACACAGGTTGCTC
41	CAL 9-R	ATCAACATGGTCGAGG
	CAL 9-F	CCGCCGATGATGAT
42	CAL 10-R	GGCTCCAAGACCAAG
	CAL 10-F	GGACTGATGTCTTGGTA
43	CAL 11-R	CCCAGGATGATCAACTG
	CAL 11-F	TTGGACTCCATCGATG
44	CAL 12-R	CAAGCAGCGTT
	CAL 12-F	CTAGTCTCCCGTTAGATG
45	CAL 13-R	CGAGAGATAGTCGCAT
	CAL 13-F	TCTTGACTGTGTGCC
46	CAL 14-R	GATCTTCCATGGTGTG
	CAL 14-F	TGGTCTGTTGTTCCG
47	CAL 15-R	GTGCTATGATCTTCTGC
	CAL 15-F	AGTTGTGACCGTCAT
48	CAL 16-R	CCAGTGAGCATACCA
	CAL 16-F	GAGATCCTTCTGCTGC
49	PKS III 1-R	CATAGAACCCACTCCAC
	PKS III 1-F	TCTCTGGCTTGTAGCA
50	PKS III 2-R	GTCTTCAGTGACGCTA
	PKS III 2-F	TGCTCACAACGATGG
51	PKS III 3-R	GCAACAGAGCACAC
	PKS III 3-F	TCTCTGGCTTGTAGCA
52	PKS III 4-R	ATTGTTCATGGAATGCTC
	PKS III 4-F	AACTATGGCGTACGAG
53	PKS III 5-R	CTCTGTTCGTGTCAC
	PKS III 5-F	CAGAGAGCAGAGAGTG
54	PKS III 6-R	CTCGATAGATCAGGACTTG
	PKS III 6-F	TGGACCGATTGTTGC
55	PKS III 7-R	AGTACAGTTGCTCGGA
	PKS III 7-F	CCACCAGATAGACAGG
56	PKS III 8-R	TTCCAGAAGACTGGAG
	PKS III 8-F	TTCTGACTGTGTGCAC
57	PKS III 9-R	AGCGCTTGTTTGTGT
	PKS III 9-F	CAGTTCATCAAGAGCTC
58	PKS III 10-R	CTGCTATGATTGTCAATC
	PKS III 10-F	CACTCCAACGTTGAGAT
59	PKS III 11-R	TCAGATGCGAACAGG
	PKS III 11-F	TCTTGAGTGCTCCATG
60	PKS III 12-R	GTTGTTGTCAGCACAG
	PKS III 12-F	CCACCAGCATGAATG

61	PKS III 13-R	CTAGAACCTGCTTGC
	PKS III 13-F	GGACGTGACATGAAGT
62	PKS III 14-R	TTGCATGAGAGAGGC
	PKS III 14-F	ATGCAGTTGGAGACGA
63	PKS III 15-R	ATGGCTGCAACAGAG
	PKS III 15-F	ACGAGGACGTGACAT
64	PKS III 16-R	TCCTTGCATGAGAGAG
	PKS III 16-F	AAGAATAGCAGCGCC
65	2OGD 1-R	CCTCACATCTCCAGC
	2OGD 1-F	ATCTCCAGTGGCAATG
66	2OGD 2-R	AACCACTATGGCAGGA
	2OGD 2-F	CCACTTGTGGCTTGA
67	2OGD 3-R	CTCTGCAATGCAGGA
	2OGD 3-F	ATCACACGCACAGGAA
68	2OGD 4-R	CACCTGGAGACGATCA
	2OGD 4-F	CACTCTTGTACGTGCC
69	2OGD 5-R	CTTGCAGTTGCTCTCAA
	2OGD 5-F	CTCGTTCTGGCATGTAG
70	2OGD 6-R	GATGGCTGAGAGCTT
	2OGD 6-F	AGCCATGATGGCAAG
71	2OGD 7-R	AACTCCAGTACGGTGA
	2OGD 7-F	TCTGGACCACGTCTT
72	2OGD 8-R	GCTTCAGAGGACATTCA
	2OGD 8-F	CTCACGCAGTCAAGA
73	2OGD 9-R	AATCTCTGGTCGAGAAC
	2OGD 9-F	ATCTCGCCACTCCAG
74	2OGD 10-R	GACAGTGCTGTTGCA
	2OGD 10-F	ATTAGGACGAGGATGAG
75	2OGD 11-R	TTCTTCCCATCCACG
	2OGD 11-F	TTCAGGCCAAGTGTT
76	2OGD 12-R	ATTTCTGAATGCCTGGG
	2OGD 12-F	ACATCTGCTGCTGGA
77	2OGD 13-R	GAGGTGCAGCATCTT
	2OGD 13-F	GGAAGCTCCAACCTC
78	2OGD 14-R	GATCCGGATCGTGATG
	2OGD 14-F	ATCCTGTCTTCGGACTT
79	2OGD 15-R	CTAGAGAGCGACGAG
	2OGD 15-F	CTGCAGCCTTCTCTT
80	2OGD 16-R	GATCCGGATCGTGATG
	2OGD 16-F	TGTGGCCTCACTCTT
81	P450 1-R	TGGCAGGTCCAAGTA
	P450 1-F	TCTTGGCCTTGGAGT
82	P450 2-R	GAGTAGCAGTGGCCA
	P450 2-F	ATCACAGCTCTGACG

83	P450 3-R	CCTACGGGGGTGTATT
	P450 3-F	CAATGCCTTCATCATGG
84	P450 4-R	TCCATGGCCGATTGT
	P450 4-F	TTGCAACCTTCACCT
85	P450 5-R	GATGACAGCACAGAGG
	P450 5-F	AACTCCTCTTGAGCC
86	P450 6-R	CCCTCTCATGAAATCGT
	P450 6-F	CTCAATCTCCACATGCTC
87	P450 7-R	TCTGACTGCTGACATAA
	P450 7-F	CAAGGATGAGGTTGTCT
88	P450 8-R	ATCGCTAGGCATCCA
	P450 8-F	GTCCTCCTCTTCGACA
89	P450 9-R	TATGTTAGGCAAGCTACC
	P450 9-F	GTGAGCAGCTCATATTG
90	P450 10-R	ATGTGATCACTCGCC
	P450 10-F	TAGCGTCCACTCGAG
91	P450 11-R	TCTGTCGAGCGTGATAT
	P450 11-F	TTCTGGAGCCTTGGAA
92	P450 12-R	AGCATCTGATGAGCTT
	P450 12-F	GTGGTTGTGGTCTCATA
93	P450 13-R	CATTTCTTCAAGGCTGC
	P450 13-F	CAAGTTCCTTCCGAGC
94	P450 14-R	GATATGCTACAGGTCTTC
	P450 14-F	TGGTGATGCACCAAC
95	P450 15-R	AAAGCAGGCAGCTAC
	P450 15-F	CTTCTCTGTGATGCCT
96	P450 16-R	CTCTGAAGCCTAGGCT
	P450 16-F	TTGAGCACAGCCTTG

F, forward; R, reverse.
